# Rational Design and Characterization of Materials for Optimized Additive Manufacturing by Digital Light Processing

**DOI:** 10.3390/polym15020287

**Published:** 2023-01-06

**Authors:** Rajat Chaudhary, Raziyeh Akbari, Carlo Antonini

**Affiliations:** Department of Materials Science, University of Milano-Bicocca, Via R. Cozzi 55, 20125 Milan, Italy

**Keywords:** digital light processing, vat photopolymerization, ceramic suspension, metal suspension

## Abstract

Additive manufacturing technologies are developed and utilized to manufacture complex, lightweight, functional, and non-functional components with optimized material consumption. Among them, vat polymerization-based digital light processing (DLP) exploits the polymerization of photocurable resins in the layer-by-layer production of three-dimensional objects. With the rapid growth of the technology in the last few years, DLP requires a rational design framework for printing process optimization based on the specific material and printer characteristics. In this work, we investigate the curing of pure photopolymers, as well as ceramic and metal suspensions, to characterize the material properties relevant to the printing process, such as penetration depth and critical energy. Based on the theoretical framework offered by the Beer–Lambert law for absorption and on experimental results, we define a printing space that can be used to rationally design new materials and optimize the printing process using digital light processing. The proposed methodology enables printing optimization for any material and printer combination, based on simple preliminary material characterization tests to define the printing space. Also, this methodology can be generalized and applied to other vat polymerization technologies.

## 1. Introduction

Additive manufacturing (AM) or 3D printing is a process, based on different technologies, for the design and fabrication of three-dimensional objects [1], an alternative to conventional subtractive manufacturing. AM enables cost-effective manufacturing, especially for complex geometries, potentially with lower environmental impacts compared to conventional processes [2], due to design flexibility and reduced waste. These advantages have drawn the attention of various industrial sectors for a variety of applications. One of the latest examples is the COVID-19 pandemic, when AM proved to be a valuable supplementary manufacturing process to align the supply chain of personal protective equipment kits in a very short time [3]. Subsequently, the AM industry grew by 20% in 2021 [4]. Currently, several AM technologies are being used to manufacture lightweight, functional, and non-functional parts, and are typically divided into the following major categories: vat polymerization, binder jetting, material jetting, powder bed fusion, direct energy deposition, material extrusion, and sheet lamination [5].

Among the different vat polymerization approaches [6], digital light processing (DLP) is one of the most recent and promising technologies for producing high-definition functional and non-functional parts [7,8]. When compared to stereolithography (SLA) [9], which is based on a laser source for voxel-by-voxel polymerization, one of the major advantages of DLP is the use of a high-resolution source [10], curing an entire layer with one projection at each step, thus reducing the overall printing time. Initially developed for producing prototypes using pure photopolymers (resins), the technology has been recently tested to print ceramic and metal suspensions [11]. One of the earliest introductions of suspensions was in the form of ceramic powder, such as alumina and zirconia, in freeform fabrication using SLA [12]. This was also extended for DLP [13], for indirect fabrication of solid complex parts with good surface quality and high precision [14], after a two-step thermal treatment: (i) debinding, for removing photopolymer matrix, and (ii) sintering, for material consolidation [15]. Similar to ceramics, metal suspensions have also been proposed for the high-speed fabrication of metal parts after thermal treatment [16]. Many companies have commercialized ceramic and metal-based DLP printers and resins for producing high-end parts [7]. Currently, the technology is being used to produce 3D parts using functional materials [17], such as elastomers [18], conductive polymers [19,20], shape memory polymers [21], biopolymers [22,23,24,25], and piezoelectric materials [26]. These photocurable, environmentally responsive materials add another dimension to the printed part, extending 3D printing to 4D printing [27,28,29].

Several advancements in radiation sources, such as liquid crystal display (LCD) [30] and projection micro stereolithography (PμSL) [31], have been proposed for layer-by-layer centered vat polymerization processes. All these technologies differ by the radiation source, and so do the irradiance, wavelength, resolution, scanning velocity, etc. that control the polymerization of the liquid photopolymer inside the vat. Moreover, radiation characteristics can be different in two printers that work on the same technology, from two different manufacturers. Therefore, it is essential to investigate how the photopolymers (pure or suspensions) are cured, e.g., by measuring the cure depth at a certain exposure time for a given wavelength, and then generalize the vat polymerization processes, to provide a framework for the printing of different materials using different printers.

The photopolymer curing depends on the composition, which typically comprises monomers, oligomers, and a suitable photoinitiator. In high-yield photopolymers, additives such as co-initiators, inert dyes, photosensitizers, and inhibitors can also be present, although it may be difficult to know the exact composition of proprietary and commercial formulations. These compositions are classified based on the photoinitiating reactions: free radicals and ionic [32]. Generally, in DLP, and specifically in this study, acrylate-based photopolymers, following a free-radical chain-growth polymerization, are used, as they are characterized by a high reactivity when exposed to UV or visible radiation [33,34]. This combines low-temperature energy-efficient DLP technology with a wide range of materials [11]. In all layer-by-layer centered vat polymerization-based printers layer thickness and exposure time are common printing parameters, so it is essential to investigate the correlation between cure depth and exposure time at a certain wavelength and irradiance. Prediction of these parameters, for a photopolymer, ensures optimum polymerization of the layers to avoid printing failure or component breakdown.

Modeling and testing photopolymerization for vat polymerization processes has been intensively performed in the literature. Jacobs [35] laid out the fundamentals of SLA using the Beer–Lambert law and provided a working curve equation,

(1) $$D_{c}=D_{p}\ln\left(\frac{E_{0}}{E_{c}}\right)$$

where D_c_ represents the cure depth of the polymer irradiated with energy E_0_. Using a logarithmic plot of D_c_ vs. E_0_, the slope and the intercept represent D_p_, the penetration depth, and E_c_, the critical exposure of radiation, respectively. These are two completely resin parameters independent of radiation power. Later, Griffith et al. [36] investigated the radiation interaction in concentrated ceramic suspensions for photopolymerization in stereolithography. They reported that the cure depth of suspensions is also affected by the size of the filler particles, scattering efficiency term, and refractive index contrast between the photopolymer and the particles. Lee et al. [37] presented the model of cure depth while changing the photoinitiator concentration. They found that there is an optimal photoinitiator concentration in the photopolymer composition to maximize the cure depth. In another model presented by Tomeckova [38], inert dye concentration was also considered with photoinitiator and ceramic concentration. They concluded that the sensitivity (inverse of cure depth) is the linear function of the photoinitiator or dye concentration. Gong et al. [39] developed a mathematical model for optical dose (irradiance × time) delivered as a function of cure depth, including voids for microfluidic applications. Further, in-situ characterization techniques are reported, such as ultrasonic imaging [40,41,42], atomic force microscopy [43], and FTIR spectroscopy [44,45] for real-time prediction of polymerization and quick optimization of parameters before printing with polymer-based additive manufacturing.

In this study, we focus on DLP to investigate the photopolymerization of pure photopolymers, as well as ceramic and metal suspensions. We propose a simple methodology based on preliminary printing tests of single layers, which can be used to identify the material properties, including the penetration depth, D_p_, and critical energy, E_c_. These properties were further investigated by varying the printing parameters, particularly the radiation power intensity. Such information is the base to draw a printing map, using layer thickness and exposure time as independent variables for printing. This proposed printing map, which represents the main novelty of this study, makes it possible to define a printing space within the constraint of material printability, thus ensuring optimal polymerization during three-dimensional object printing.

## 2. Materials and Methods

### 2.1. Photopolymers

Tests were conducted using the following five commercial photopolymers: G-Strong (Sharebot S.r.l., Nibionno, Italy), Model Resin (HARZ Labs LLC., Moscow, Russia), Dental Clear (HARZ Labs LLC., Moscow, Russia), Porcelite (Tethon 3D, Omaha, NE, USA), and Ferrolite (Tethon 3D, Omaha, NE, USA). G-strong, Model Resin, and Dental Clear are the mixtures of (meth-) acrylate-based monomers, oligomers, and photoinitiator (undefined) with different color dyes: grey, black, and translucent, respectively. Porcelite and Ferrolite are alumina- and iron-based suspensions, respectively, with similar compositions to pure photopolymers. Powder concentration was found to be 52% *w*/*w* and 57% *w*/*w* in Porcelite and Ferrolite, respectively, with particles in the range of 10–40 µm (See Supplementary Materials, Figure S1 for detailed information on TGA and Figure S2 for particle size imaging). All materials were used as received, except Ferrolite, to which 1% of diphenyl(2,4,6-trimethylbenzoyl) phosphine oxide (TPO) (purity > 98.0%) (TCI Europe N.V., Zwijndrecht, Belgium) was added as a photoinitiator. All the resins underwent chain polymerization through a free radical mechanism.

### 2.2. Printer and Software

**Figure 1:**
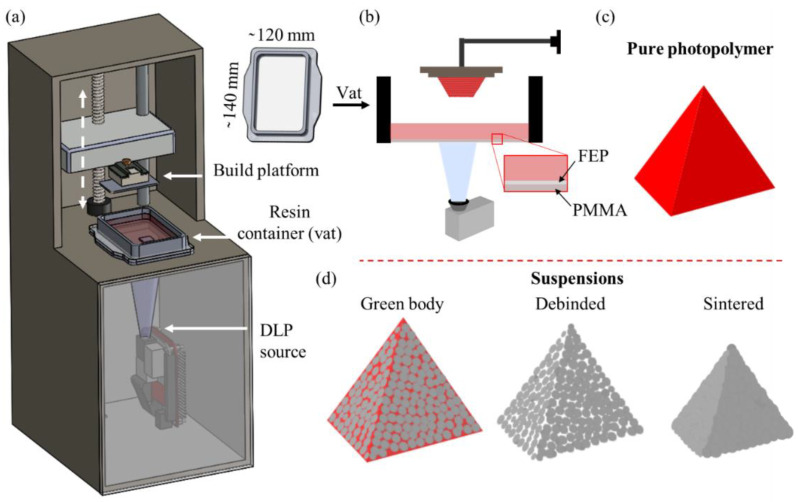
CAD model (**a**) and schematic (**b**) of a bottom-up DLP printer enabling the manufacturing of 3D objects from pure photopolymers (**c**) and suspensions followed by debinding, for thermal decomposition and removal of photopolymer, and sintering, for powder consolidation (**d**).

A commercially available DLP printer, Voyager Z-20 from Sharebot (Figure 1a), was used in the study. The printer is equipped with an LED-powered DLP source, with a printing resolution (i.e., pixel size) of 50 μm over a projection window of 99.8 × 56.1 mm^2^ and a vat container of area ~140 × 120 mm^2^. This projection enables layer-by-layer polymerization for the printing of three-dimensional objects, using an inverted bottom-up configuration, with the projector positioned below the vat (Figure 1b). A fluorinated ethylene propylene (FEP) film over polymethyl methacrylate (PMMA) facilitates the soft detachment of each polymerized layer from the transparent vat base, due to the low-surface energy interface. This printer can be used for object fabrication, starting from either pure photopolymer (Figure 1c) or suspension, which can, eventually, be thermally treated during debinding and sintering (Figure 1d).

Printing parameters, including layer thickness, exposure time, irradiance, and layer interface separation time, were regulated by printer-specific Pyramis software (Sharebot) specifically configured for this printer.

### 2.3. Sample Preparation

When the resin is exposed to radiation for a given polymerization time, t_p_ (corresponding to a given radiated energy, E_0_), the resin polymerizes to form a film with a layer thickness, D_c_. To study the correlation between D_c_ and t_p_ (or E_0_), preliminary photopolymerization tests were conducted to produce polymerized films in a free resin bath, in absence of the printing head (referred to as “no printing head” configuration). A single-layer CAD file to print a film with an area of 15 × 15 mm^2^ (an arbitrary height of 50 μm was defined for the film thickness) was generated in Autodesk Fusion 360 and subsequently converted as STL as input for the Pyramis software. The vat container was filled with enough resin (typically up to 5 mm), ensuring that the resin at the bottom was not exposed to air, to avoid oxygen inhibition during polymerization. Fifteen monolayer films with different thicknesses—using different irradiation times from 1 s to 8 s, in increments of 0.5 s—were prepared with each resin. Tests were then systematically repeated, modulating the radiation power intensity at 100% (maximum), 80%, 60%, and 40% (see the result between intensity and irradiance in Section 3.1), to investigate the effect of radiation power on polymerization (corresponding to sample sets #1 to #4, respectively, Table 1). These polymerized monolayer films were carefully detached from the vat to measure the thickness.

For FTIR analysis, monolayer films were prepared using the G-Strong resin in no printing head configuration. The same CAD file and procedure were followed as for previous sample sets, irradiating from 1 s to 8 s in increments of 1 s, at 100% intensity (sample set #5, Table 1). FTIR measurements were performed on the UV-exposed side of the monolayer films. Polymerized monolayer films were extracted from the vat after removing the liquid photopolymer, to avoid contamination of the exposed side to the liquid photopolymer.

### 2.4. Characterization

#### 2.4.1. Radiation Characterization

The radiation spectrum of the DLP source was analyzed by a mini-spectrometer (C10083CA, Hamamatsu, Japan) with a spectral resolution (FWHM) of 5 nm. The radiation was collected using an optical fiber directed in the center of the projection area on the vat.

To investigate irradiance at different grayscales of projection, a power meter (PM200, Thorlabs, Jessup, MD, USA) with Si photodiode power sensor (S120VC Thorlabs, US; aperture diameter 9.5 mm, measurement uncertainty ± 5%) was used. The measurements were taken while setting the attenuation to 0 dB, wavelength 405 nm, bandwidth 10 kHz, and range 18.0 mW in the power meter. The photodiode was placed at five different points on the vat, at the center of the projection area and at each of the four corners, to verify irradiance homogeneity in the plane of projection.

#### 2.4.2. Material Characterization

The UV-Vis absorption of the photopolymers was collected using a UV-Vis spectrophotometer (Cary 60, Agilent, Santa Clara, CA, USA). The scan was performed in the range of 200–800 nm, with a scan rate of 600 nm/min. The liquid photopolymer samples (thickness: 0.12 ± 0.01 mm) were sandwiched between two microscopic glass slides (thickness: 1 mm).

The polymerized monolayer film thickness was measured using a digital caliper (2972, Kraftwerk, Zurich, Switzerland) with a resolution of 0.01 mm (accuracy: ±0.02 mm for size < 100 mm), after gentle removal of excess liquid photopolymer with tissue paper. The average and standard deviation were computed based on four measurements, taken on each side of the squared layer.

The IR absorption of polymerized monolayer films was recorded by the FTIR spectrometer (JASCO 4100, Easton, MD, USA) using an attenuated transmission reflectance (ATR) accessory equipped with a ZnSe crystal. After careful extraction from the vat, the polymerized monolayer films were placed on the ATR crystal from the UV-exposed sides and scanned at three different points. The transmittance was recorded in the range 550–4000 cm^−1^, using 4 cm^−1^ resolution after 64 scans.

Rheology measurements were performed with a rotational rheometer, MCR 92 (Anton Paar, Graz, Austria), using a 50 mm parallel plate setup. Rotational tests were carried out to determine the viscosities of all the photopolymers and suspensions, using a plate gap of 0.25 mm and a linear ramp shear rate of 0–100 s^−1^ at 24 °C.

### 2.5. Light-Matter Interaction

#### 2.5.1. Pure Resin

The free radical polymerization process, generally for acrylates, is most widely utilized in vat polymerization for AM due to higher reactivity and yield, which makes the process effective. The radical generation is promoted by the photoinitiator, as the consequence of bond cleavage and hydrogen abstraction, during the initiation process under exposure to radiation. The polymerization then starts and proceeds with monomers and oligomers forming longer chains. Later, the reaction is terminated due to one of the following reasons: (ⅰ) joining of two polymer chains containing free radicals (recombination), (ⅱ) the cancellation of one free radical by another without joining (disproportion), or (ⅲ) the trapping of free radicals inside the polymeric chain (occlusion) [46].

The characteristics of a photopolymer before, during, and after curing rely on the composition. The ratio between oligomers and monomers determines the viscosity of the mixture and affects the final mechanical properties of the printed object. The photoinitiator concentration is crucial for the correct polymerization: an optimal concentration can typically be defined to maximize the polymerization depth for AM [37]. Moreover, the photopolymerization rate is related to the monomer concentration decrease, which is given by the sum of the initiation rate, R_i_, and the propagation rate, R_p_, with R_p_ >> R_i_, thus,

(2) $$\mathrm{Polymerization}\;\mathrm{rate}=\frac{-d\left[M\right]}{dt}=R_{i}+R_{p}\sim R_{p}$$

The polymerization rate (or propagation rate, R_p_) can be expressed as the sum of all individual propagation rates, which are the same for all growing chains,

(3) $$R_{p}=k_{p}\left[M\right]\left[M^{\prime }\right]$$

where k_p_ is the propagation rate constant, and [M] and [M′] are the concentration of monomers and all growing chains, respectively [47]. The equation cannot be used directly as the concentration of growing chains is difficult to measure. Therefore, a steady-state assumption is made, where the number of chains grows rapidly initially and reaches a steady state, and the rate of change quickly becomes zero. This implies that in steady-state conditions the initiation and termination rates are equal, i.e.,

(4) $$R_{i}=R_{t}=2k_{t}{\left[M^{\prime }\right]}^{2}$$

where k_t_ is the termination rate constant. Substituting the value of [M′] in Equation (3),

(5) $$R_{p}=k_{p}\left[M\right]{\left(\frac{R_{i}}{2k_{t}}\right)}^{\frac{1}{2}}$$

For photoinitiated reactions, the initiation rate can be expressed as

(6) $$R_{i}=2\Phi I_{V}$$

where Φ is the quantum yield of the photoinitiator, I_V_ denotes the photons (in moles) absorbed per unit volume and time (Einstein or mol cm^−3^ s^−1^), and the numeric factor 2 denotes the number of generated free radicals during photolysis. Substituting into Equation (5),

(7) $$R_{p}=k_{p}\left[M\right]{\left(\frac{\Phi I_{V}}{k_{t}}\right)}^{\frac{1}{2}}$$

Beer–Lambert’s law can be used to determine the absorbed light as

(8) $$I_{S}=I_{0}\left(1-e^{-\alpha \left[PI\right]z}\right)$$

where I_S_ and I_0_ (both are surface light intensity) are the absorbed light at distance z and on the surface, respectively. [PI] and α are the molar concentration and absorption coefficient of the photoinitiator.

To determine the absorbed light intensity, I_V_, at the distance z inside the vat, the surface intensity, I_S_, can be differentiated with respect to z,

(9) $$I_{V}=\frac{dI_{S}}{dz}=\alpha \left[PI\right]I_{0}e^{-\alpha \left[PI\right]z}$$

Substituting I_V_ in Equation (7),

(10) $$R_{p}=k_{p}\left[M\right]{\left(\frac{\Phi \alpha \left[PI\right]I_{0}e^{-\alpha \left[PI\right]z}}{k_{t}}\right)}^{\frac{1}{2}}$$

The term represents the polymerization rate at distance, D_c_. Further, from Equation (2),

(11) $$\frac{-d\left[M\right]}{dt}=R_{p}=k_{p}\left[M\right]{\left(\frac{\Phi \alpha \left[PI\right]I_{0}e^{-\alpha \left[PI\right]z}}{k_{t}}\right)}^{\frac{1}{2}}$$

Separating variables and integrating with the assumption of no time dependency in the bracketed term on the right-hand side gives,

(12) $$ln\frac{{\left[M\right]}_{0}}{\left[M\right]}={\left(\frac{k_{p}{}^{2}\Phi \alpha \left[PI\right]I_{0}e^{-\alpha \left[PI\right]z}}{k_{t}}\right)}^{\frac{1}{2}}\cdot t$$

The term on the left-hand side is simply the degree of polymerization with monomer conversion from [M]_0_ to [M] after certain exposure (t = t_p_),

(13) $$\mathrm{Degree}\;\mathrm{of}\;\mathrm{polymerization}\left(x\right)=\frac{{\left[M\right]}_{0}}{\left[M\right]}=\frac{1}{1-p}$$

where p is the extent of polymerization. At the gel point, p = p_c_, the critical threshold for gelation. It, therefore, corresponds to the limit of the cure depth (z = D_c_) in the photocuring process and is a characteristic of the photochemical system. From Equations (12) and (13),

(14) $$\left[\frac{k_{t}}{k_{p}{}^{2}\Phi \alpha I_{0}}\right]{\left[\frac{ln\left(1-p_{c}\right)}{t_{p}}\right]}^{2}=\left[PI\right]e^{-\alpha \left[PI\right]D_{c}}$$

Or,

(15) $$\left[\frac{k_{t}{\left\{ln\left(1-p_{c}\right)\right\}}^{2}}{k_{p}{}^{2}\Phi \alpha }\right]\frac{1}{I_{0}t_{p}^{2}}=\left[PI\right]e^{-\alpha \left[PI\right]D_{c}}$$

In DLP, the liquid photopolymer is polymerized by modulated light, which is reflected from DMD and focused on the vat base by an objective lens. Several models have been introduced to standardize the curing with pixel-based systems, assuming the reflected light from the single micromirror is incoherent but follows Gaussian distribution [48], as this is typical for lasers used in SLA. However, for our purpose, such assumption is not necessary, and we can simply consider an average irradiance within the illuminated pixel, I_av_ (W/cm^2^), so that the energy per unit area at the vat base can be expressed as,

(16) $$E_{0}=I_{av}\cdot t_{p}=\left(\frac{Nhc}{\lambda }\right)I_{0}\cdot t_{p}$$

Rearranging and substituting values of I_0_ and t_p_ in Equation (15) gives

(17) $$\left[\frac{k_{t}{\left\{ln\left(1-p_{c}\right)\right\}}^{2}}{k_{p}{}^{2}\Phi \alpha }\right]\frac{NhcI_{av}}{\lambda E_{0}^{2}}=\left[PI\right]e^{-\alpha \left[PI\right]D_{c}}$$

Substituting $\left[\frac{k_{t}{\left\{ln\left(1-p_{c}\right)\right\}}^{2}}{k_{p}{}^{2}\Phi \alpha }\right]=A^{2}$, parameters based on the composition of the resin, $\frac{NhcI_{av}}{\lambda }=B^{2}$, light parameters, and solving Equation (17),

(18) $$D_{c}=\frac{2}{\alpha \left[PI\right]}ln\left[\frac{E_{0}{\left[PI\right]}^{\frac{1}{2}}}{AB}\right]$$

By comparing the above equation with Jacob’s fundamental Equation (1) derived for stereolithography, one recognizes that the penetration depth, D_p_, depends on the photoinitiator concentration, whereas critical energy is determined by both the photoinitiator concentration and the radiation source [37].

Furthermore, for a constant radiation rate, the above equation can be rewritten as [39],

(19) $$D_{c}=D_{p}\ln\left(\frac{t_{p}}{t_{c}}\right)$$

where $t_{c}=E_{c}/I_{av}$ is the so-called critical time needed to start polymerization. This equation is another representation of Equation (1), under a constant radiation condition.

#### 2.5.2. Suspensions

The above derivation can, in principle, be extended from the case of pure resins to ceramic and metal suspensions. Generally, highly reactive acrylate-based compositions are the primary choice for the inclusion of micro- and nano-sized suspensions. Moreover, the reaction mechanism is not usually affected in the presence of particles; however, light-matter interaction is affected due to the scattering and absorption of the irradiation, which results in the reduction of irradiation penetration. In early findings, Griffith et al. [36] remodeled the fundamental equation of stereolithography for scattering in turbid ceramic suspensions as follows,

(20) $$D_{c}=\frac{2\langle d\rangle }{3\tilde{Q}}\frac{1}{\phi }\frac{n_{0}^{2}}{{\left(n_{p}-n_{0}\right)}^{2}}\ln\left(\frac{E_{0}}{E_{c}}\right)$$

where <d> represents average particle size, $\tilde{Q}$ the scattering coefficient, ϕ the powder fraction, and n_0_ and n_p_ the refractive indexes of liquid polymer and powder, respectively.

Therefore, in suspensions, the cure depth depends not only on the light characteristics and polymer composition, but also on the particle size, scattering coefficient, powder fraction, and refractive index of pure photopolymer and powder. Cure depth improves in the compositions, having: (i) minimal difference in photopolymer and particles refractive index, (ii) low powder fraction with big-sized particles, and (iii) high scattering coefficient. However, manufacturing objects with precise geometry with high density requires high loading with small particle size, which leads to a decrease in the cure depth and, also, an increase in the viscosity. The problem is countered by the optimization of the composition by adding other components, such as inhibitors to reduce the absorbance, which increases the required radiation dose [49].

## 3. Results and Discussion

### 3.1. Radiation Source Characteristics

**Figure 2:**
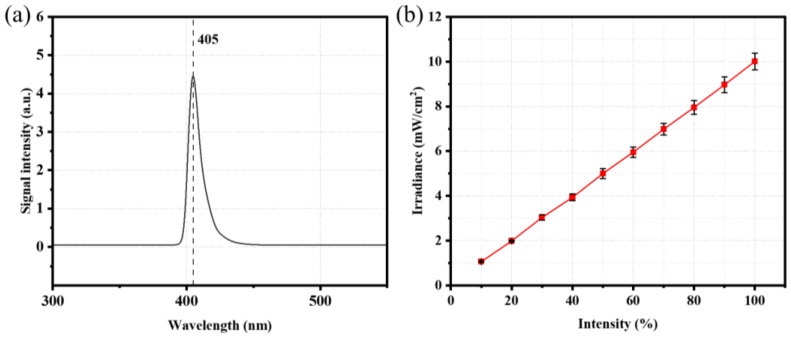
UV light characteristics of DLP printer: (**a**) radiation spectrum of the source, (**b**) irradiance at different light intensities (grayscale).

The radiation spectrum of the DLP source, illustrated in Figure 2a, presents a peak at 405 nm, as expected by manufacturer information. As such, photopolymers need to absorb radiation around this wavelength to be photopolymerizable and printable. With respect to irradiance, preliminary tests confirmed a linear relationship between the projected light intensity (%) and irradiance, see Figure 2b, as measured at five different points on the vat base.

### 3.2. Preliminary Characterization of Resins

**Figure 3:**
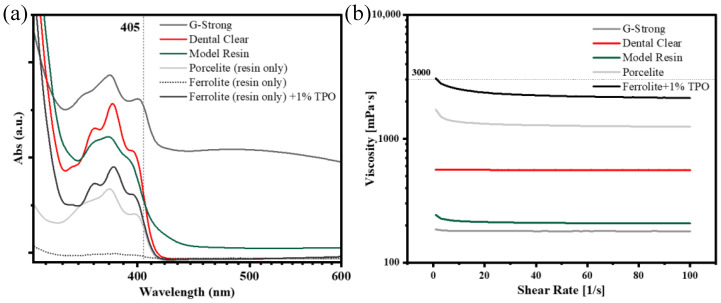
Characterization of photopolymers: (**a**) UV-Vis absorption and (**b**) rheology of the photopolymers and suspensions. Dashed lines in (**a**,**b**) correspond to the wavelength of the DLP source and reported limit of viscosity for self-recoating of liquid photopolymer, respectively.

The compatibility of photopolymers with the DLP source was validated by preliminary UV-Visible spectroscopy analysis. All the pure resins—G-Strong, Model Resin, and Dental Clear—showed absorbance at 405 nm, corresponding to the peak wavelength of the DLP radiation (see Figure 3a). For suspensions, the base resins of Porcelite and Ferrolite were tested after particle sedimentation. The base resin of Porcelite shows good absorption close to 405 nm, whereas the Ferrolite base resin presents no significant peak. As such, 1 wt% of TPO, a photoinitiator, was added to Ferrolite, significantly increasing the absorption and, thus, the photopolymerization (see Figure 3a).

Rheology measurements confirm that all resins can be considered Newtonian fluids, as the viscosity is constant in the investigated shear rate range (see Figure 3b). Viscosity is in the order of 10^2^ mPa·s for pure resins and 10^3^ mPa·s for suspensions. Nonetheless, all viscosities were found to be lower than the threshold of 3000 mPa·s; this value is considered in the literature as an upper limit for the liquid resin viscosity, to ensure full recoat of the vat base and, thus, good adhesion between two consecutive printing steps [10].

### 3.3. Polymerization of Monolayer Films

#### 3.3.1. Pure Photopolymers

**Figure 4:**
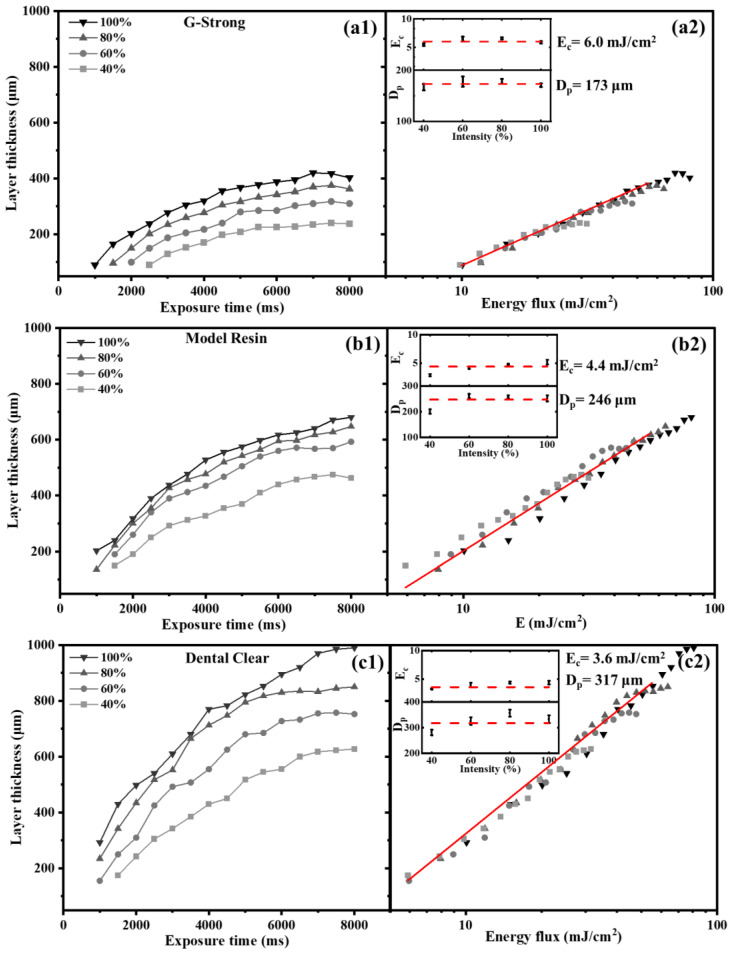
Monolayer film thickness as a function of exposure time at different light intensities (**a1**,**b1**,**c1**) and as a function of radiated energy flux (**a2**,**b2**,**c2**), for determination of critical energy (E_c_) and penetration depth (D_p_) of pure resins: G-Strong (**a1**,**a2**), Model Resin (**b1**,**b2**) and Dental Clear (**c1**,**c2**).

The correlation between exposure time and the polymerized layer thickness is demonstrated by polymerizing the monolayer films at different exposure times in no printing head configuration. The variation of polymerized layer thickness with exposure time is shown in Figure 4(a1,b1,c1) for the pure photopolymers, G-Strong, Model Resin, and Dental Clear, respectively. The different curves in each graph correspond to different radiation intensities, which were modulated from 40 to 100% (corresponding to irradiance values in the range of 4 to 10 mW/m^2^; see Figure 2b). As expected, the monolayer film thickness increases with both exposure time and irradiance. Also, a monolayer film forms after a certain critical time, which decreases with increasing irradiance. Since the product between the exposure time and irradiance is the energy flux, E_0_, the thickness values are also presented in Figure 4(a2,b2,c2) as a function of the energy, using a semi-logarithmic plot. For each material, data clearly collapse on a single master curve, which can be fitted using Equation (1) (Jacob’s law), to identify experimentally two values: penetration depth and critical energy. For the fitting, only the experimental data below saturation (~50–60 mJ/cm^2^) are selected. Insets in Figure 4(a2,b2,c2) show D_p_ and E_c_ values as a function of light intensity (i.e., irradiance), with the red dashed curve representing the value obtained by the collective data average: for G-Strong, 173 μm and 6 mJ/cm^2^; for Model Black, 246 μm and 4.4 mJ/cm^2^; and for Dental Clear, 316 μm and 3.6 mJ/cm^2^, respectively.

#### 3.3.2. Suspensions

**Figure 5:**
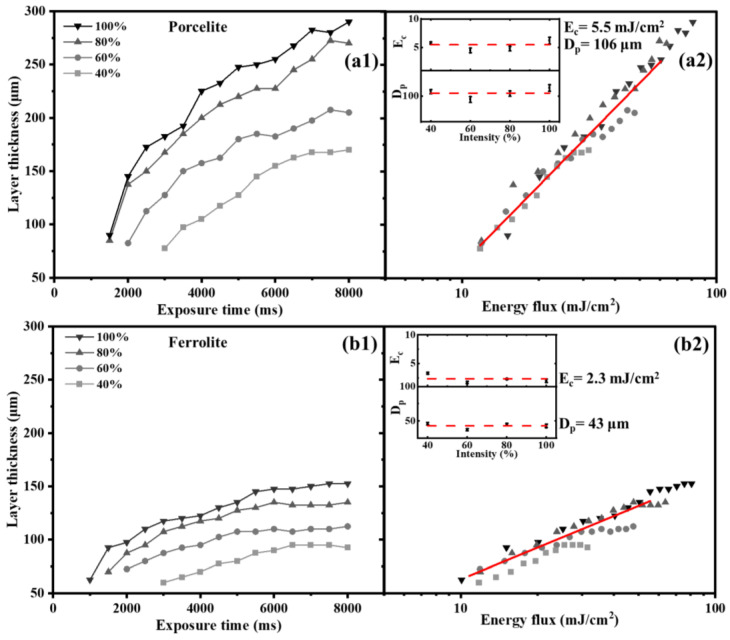
Monolayer film thickness as a function of exposure time at different light intensities (**a1**,**b1**) and as a function of radiated energy flux (**a2**,**b2**), for determination of critical energy (E_c_) and penetration depth (D_p_) of pure suspensions: Porcelite (**a1**,**a2**) and Ferrolite (+1% TPO) (**b1**,**b2**).

The study was also extended to two suspensions, Porcelite and Ferrolite, with results illustrated in Figure 5. Similar trends to pure photopolymers were observed, with the monolayer film thickness increasing with both exposure time and irradiance, and the formation of a layer after a critical time (see Figure 5(a1,b1)). Even for each suspension, data collapse on a single master curve, to quantify penetration depth and critical energy. Experimental data below saturation are used for fitting, using Jacob’s equation (see Figure 5(a2,b2)), to find two characteristic parameters, D_p_ and E_c_: for Porcelite, 106 μm and 5.5 mJ/cm^2^, and for Ferrolite, 43 μm and 2.3 mJ/cm^2^, respectively. Clearly, highly concentrated suspensions have low irradiation penetration due to scattering and absorption by the suspended powder (see Section 2.5.2). This reduces the penetration depth of the irradiation.

### 3.4. FTIR-ATR Analysis

**Figure 6:**
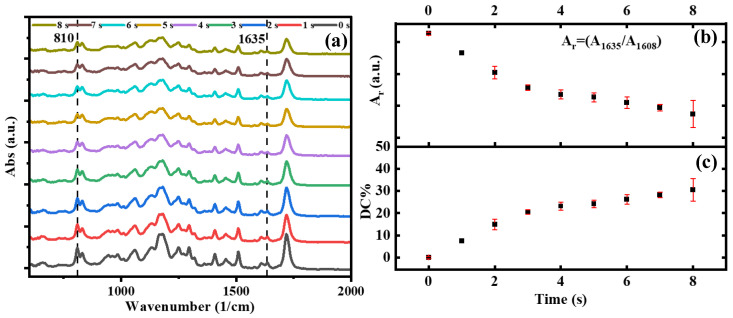
FTIR-ATR absorption spectra of freely polymerized layers and unpolymerized resin (**a**), relative absorbance (**b**), and degree of double bond conversion (**c**) of monolayer films polymerized at different exposure times without printing head configuration.

FTIR-ATR spectroscopy was performed to understand the curing behavior of photopolymers with exposure times. Figure 6a shows the absorption spectra of eight monolayer films prepared with G-Strong resin, each exposed to radiation from 1 to 8 s, with 1 s interval increases (sample set #5), and the absorption spectrum of the original unexposed resin (0 s). Several vibrational peaks can be investigated to evaluate the conversion of acrylates such as CH=CH_2_ twisting at 810 cm^−1^, C-O stretching at 1192 cm^−1^, CH_2_ scissor deformation at 1405 cm^−1^, and CH_2_=CH_2_ stretching at 1635 cm^−1^, as shown in the literature [44]. The region between 900–1600 cm^−1^ (see Figure 6a) was the result of the overlap of several bands due to different bending modes. Both absorption peaks at 1635 cm^−1^ and 810 cm^−1^ decrease with increasing exposure time, as a consequence of the decrease in the double bonds (C=C) of the acrylate group during polymerization. Among the two peaks, based on previous similar studies in the literature [50,51], we selected the absorption peak at 1635 cm^−1^ to quantify the degree of conversion during polymerization. Also, for our measurement, this peak shows a clear trend, compared to the peak at 810 cm^−1^, which is more subject to noise [52]. The absorbance peak for aromatic at 1608 cm^−1^, corresponding to C=C stretching, was chosen as the reference peak for normalization. Thus, the conversion was calculated as,

(21) $$\mathrm{Degree}\;\mathrm{of}\;\mathrm{Conversion}\;\left(DC\right)\left(\%\right)=\frac{{\left(\frac{A_{1635}}{A_{1608}}\right)}_{0}-{\left(\frac{A_{1635}}{A_{1608}}\right)}_{t}}{{\left(\frac{A_{1635}}{A_{1608}}\right)}_{0}}\times 100$$

where the subscripts 0 and t refer to time. The relative absorbance, (A_1635_/A_1608_)_t_, decreases for increasing exposure time (see Figure 6b) and, correspondingly, the degree of conversion increases to ~30% after 8 s of exposure. The increase at initial exposures shows the rapid growth in polymeric chains while consuming the reactive species and reaching a steady state.

One should note that the polymerization is not homogenous within the bulk, as the radiation decays exponentially from the surface to the inner part of the material (see Equation (8)) [43]. Therefore, the degree of polymerization is not the same on the two sides of the polymerized layer, the bottom side (which is directly exposed to radiation) and the top side; the difference generally depends on the thickness. We have also performed additional tests to compare the difference in the degree of polymerization between the two sides, but they have not been reported due to lack of consistency; the main practical issue in such analysis is that the top layer remains wet by the unpolymerized resin. Both direct measurements of the top layer, taken without cleaning and after cleaning with solvent, do not provide clear information on the surface composition during FTIR tests. Nonetheless, from an application point of view, sufficient polymerization should be ensured along the entire thickness, from the bottom to the top side, to facilitate good adhesion with the printing head and between consecutive layers. A decrease in the layer thickness at each step has to be defined for minimizing the difference in the polymerization along the thickness. Thus, a compromise needs to be found between homogeneity, required resolution, and overall printing time.

### 3.5. Optimized 3D Printing Space

**Figure 7:**
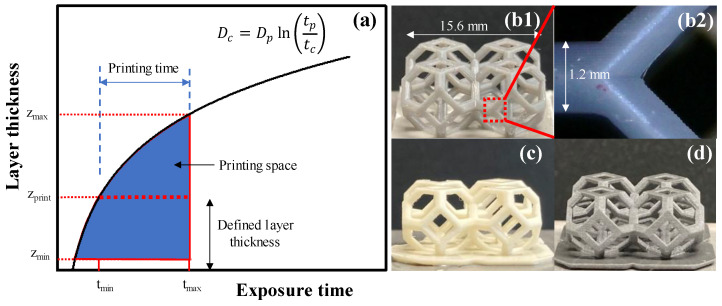
Schematic of DLP printing map defining optimized printing space (**a**). Objects printed with G-Strong (**b1**), (**b2**) Porcelite (**c**), and Ferrolite (**d**) resin using the parameters from the map.

The trends observed for layer thickness increase as a function of the exposure time, enabling the definition of a 3D printing map (illustrated in Figure 7a), where an optimal 3D printing space can be specified based on simple preliminary experiments, such as those presented above.

Using the exposure time and the layer thickness as primary parameters and assuming a given constant irradiation, I_av_, the printing space is constrained on the left by the characteristic curve defined by Jacob’s law expressed using time (see Eq. 19), and on the right by a maximum time (t_max_). This second limit is related to the polymerization of the bottom layer: since the adhesion between the vat base and the photopolymerized layer increase with the exposure time, when t_max_ is exceeded, the adhesion becomes too high, with a risk of damage for the layer or for the vat base when separating the two. At this point, the user can define the desired layer thickness, z_print_. According to the printing space, the printing time must be chosen, either t_min_ or t_max_. The minimum time, t_min_, is identified graphically as the intersection between the line corresponding to Equation (19) and the set value, z_print_ (see Figure 7a). Mathematically, this corresponds to:

(22) $$t_{min}=t_{c}e^{\frac{z_{print}}{D_{p}}}=\frac{E_{c}}{I_{av}}e^{\frac{z_{print}}{D_{p}}}$$

where E_c_ and D_p_ are the critical energy and the penetration depth of the specific material that needs to be printed. Note that the z_print_ results are constrained between a minimum, z_min_, and a maximum, z_max_, value. The minimum, z_min_, is set by the resolution of the machine (i.e., the precision of the step motor controlling the vertical motion of the printing head); also, in case of suspensions, z_min_ cannot be lower than the particle size. The maximum, z_max_, is identified graphically as the intersection between the line corresponding to Equation (19) and the maximum time, t_max_ (see Figure 7a). Indeed, according to Equation (19), layers thicker than z_max_ would require an exposure time higher than t_max_, to ensure polymerization along the entire thickness. As such, it would be impossible to satisfy both constraints ($t_{min}<t_{p}<t_{max}$) simultaneously.

Based on the above results, we demonstrate the potential of DLP to print complex objects using G-Strong (Figure 7(b1,b2)), Porcelite (Figure 7c) and Ferrolite (Figure 7d). For G-Strong and Porcelite, the layer thickness, z_print_, was set as 50 μm, which is well below the penetration depth (173 μm and 106 μm, respectively) (See Supplementary Materials, Figure S3 for SEM images of layer-by-layer structure of printed objects). For this thickness, the corresponding values for t_min_ and t_max_ for G-Strong are 0.8 s and 3.6 s; as such, an exposure time of 1.9 s was selected for printing. For Porcelite, the corresponding t_min_ and t_max_ are 0.9 s and 5 s, respectively; as such, an exposure time of 3.6 s was selected for printing. For Ferrolite, the layer thickness was set as 40 μm, which is close to the penetration depth (43 μm). For this thickness, the corresponding t_min_ and t_max_ are 0.3 s and 6 s; as such, an exposure time of 3.8 s was selected for printing. The printing of objects outside the printing space is further demonstrated in Supplementary Materials (Figure S4).

The selected exposure time, t_p_, was found adequate for polymerization and good adhesion of two consecutive printing layers. Note that, to ensure good adhesion to the printing head, only the first five layers—as base layers—have been printed with an exposure time equal to t_max_. As such, the 3D printing map, based on material characterization to define printing properties, was proved to be a valuable tool for optimal printing using digital light processing.

## 4. Conclusions

In this study, we have investigated the photopolymerization process using digital light processing (DLP), a vat polymerization-based technology for layer-by-layer production of three-dimensional objects. Specifically, we have investigated the curing of pure photopolymers, as well as ceramic and metal suspensions, to characterize the material properties relevant to the printing process, such as penetration depth and critical energy. We have shown that, on the basis of the theoretical framework offered by the Beer–Lambert law, to understand the radiation absorption within the photopolymer and the associated photopolymerization dynamics, and on the experimentally derived material properties, it is possible to define an optimal printing space. In practice, for any given material and printer combination, the methodology developed in this study allows defining a printing space where the constraints are identified starting from the experimentally measured penetration depth and critical energy and simple design principles. Such a printing space can be used to rationally design new materials and optimize the printing process using digital light processing and can be also generalized for the optimization of other vat polymerization technologies. Thus, the proposed methodology provides the basis for high-resolution printing using pure photopolymers and suspensions, including metals and ceramics, using DLP technology. In the future, thermal treatments of printed objects using ceramic- and metal-based suspensions need to be conducted to optimize both debinding and sintering, addressing material final properties.

## Figures and Tables

**Table 1:** Details of five sets of prepared samples (i.e., monolayer films, using the “no printing head” configuration; see text for details). In sets #1–#4, samples were produced for each resin, modulating the radiation intensity; in set #5, G-Strong samples were prepared for FTIR analysis.

Set	Intensity (%)	UV Exposure Time (s)	Time Step (s)	Total No. of Samples	Characterization	Note
#1	100	1–8	0.5	15	D_c_ vs. t_p_	No printing head; for each resin
#2	80	1–8	0.5	15	D_c_ vs. t_p_	No printing head; for each resin
#3	60	1–8	0.5	15	D_c_ vs. t_p_	No printing head; for each resin
#4	40	1–8	0.5	15	D_c_ vs. t_p_	No printing head; for each resin
#5	100	1–8	1	8	FTIR	No printing head; for G-Strong only

## Data Availability

The authors confirm that the data supporting the findings of this study are available within the article and Supplementary Materials. Additional data can be made available upon request.

## Supplementary Materials

The following supporting information can be downloaded at: https://www.mdpi.com/article/10.3390/polym15020287/s1, Figure S1. TGA curves for suspensions powder loading determination: Porcelite (black) and Ferrolite (red); Figure S2. Microscopic images taken for particle size determination of Porcelite (a1), (b1) and (c1), and Ferrolite (a2), (b2) and (c2); Figure S3. SEM images showing the layer-by-layer structure of G-Strong (a), Porcelite (b) and Ferrolite (c); Figure S4. Printing of the samples outside the printing space. The sample printed with G-Strong showed detachment from the print head during printing (a). Also, a reduction of exposure time for Porcelite (b) and Ferrolite (c) did not allow the base layers to adhere to the print base.
