# Supplementary material for: Rational Design and Characterization of Materials for Optimized Additive Manufacturing by Digital Light Processing
Source: Polymers (Basel). 2023 Jan 6;15(2):287. doi: 10.3390/polym15020287 (PMC9866493; doi:10.3390/polym15020287)
Supplement: Supplementary file 1 [file polymers-15-00287-s001.zip › polymers-2092559-supplementary.pdf]

Supplementary Materials

Rational Design and Characterization of Materials for Optimized Additive Manufacturing by Digital Light Processing

Rajat Chaudhary, Raziyeh Akbari and Carlo Antonini *

Department of Materials Science, University of Milano-Bicocca, Via R. Cozzi 55, 20125 Milan, Italy
* Correspondence: carlo.antonini@unimib.it

Thermogravimetric analysis

The thermogravimetric analysis (TGA) was performed to assess the powder loading for the resins Porcelite (ceramic suspension) and Ferrolite (metallic suspension). Tests with a Mettler Toledo TGA/DSC1 Star-e System. The variation of weight is analyzed by heating from 30 to 800 °C, at 10°C/min, under 50 ml/min of nitrogen flow.

The TGA degradation curves in **Error! Reference source not found.** indicate a weight loss, corresponding to the organic component in the suspension, equal to 48% and 43% for Porcelite and Ferrolite, respectively. As such, we can estimate the powder loading to be equal to 52% (Porcelite) and 57% (Ferrolite).

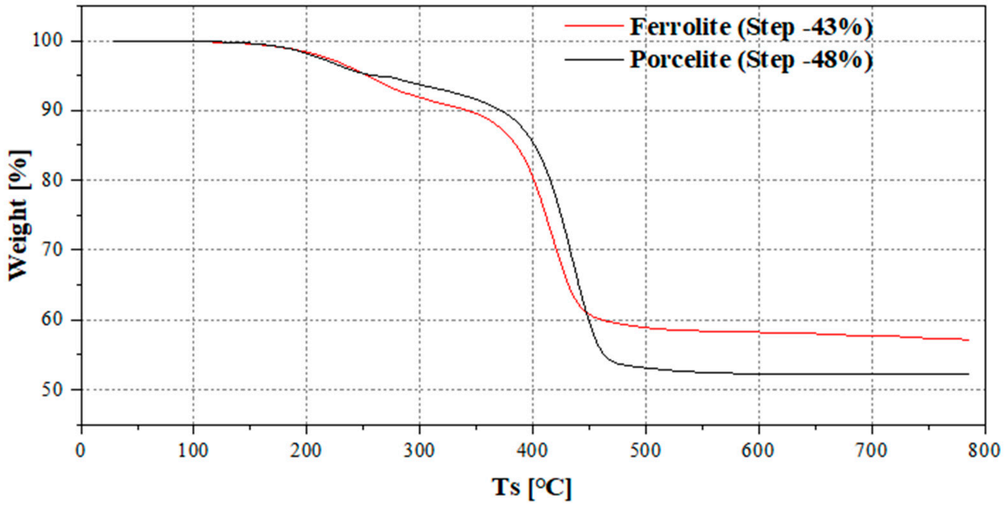

Figure S1. TGA curves for suspensions powder loading determination: Porcelite (black) and Ferrolite (red).

Optical microscope images for particle size determination

Particle size analysis for the ceramic and metallic powder in the resins Porcelite and Ferrolite was performed by optical microscopy. The optical microscope used for this research was a Leica Microsystems DM/LP equipped with 5 lenses from 5x to 1000x. Representative optical images are reported in **Error! Reference source not found.**, showing that individual particles are in the range of 10-40 μm for both Ferrolite and Porcelite.

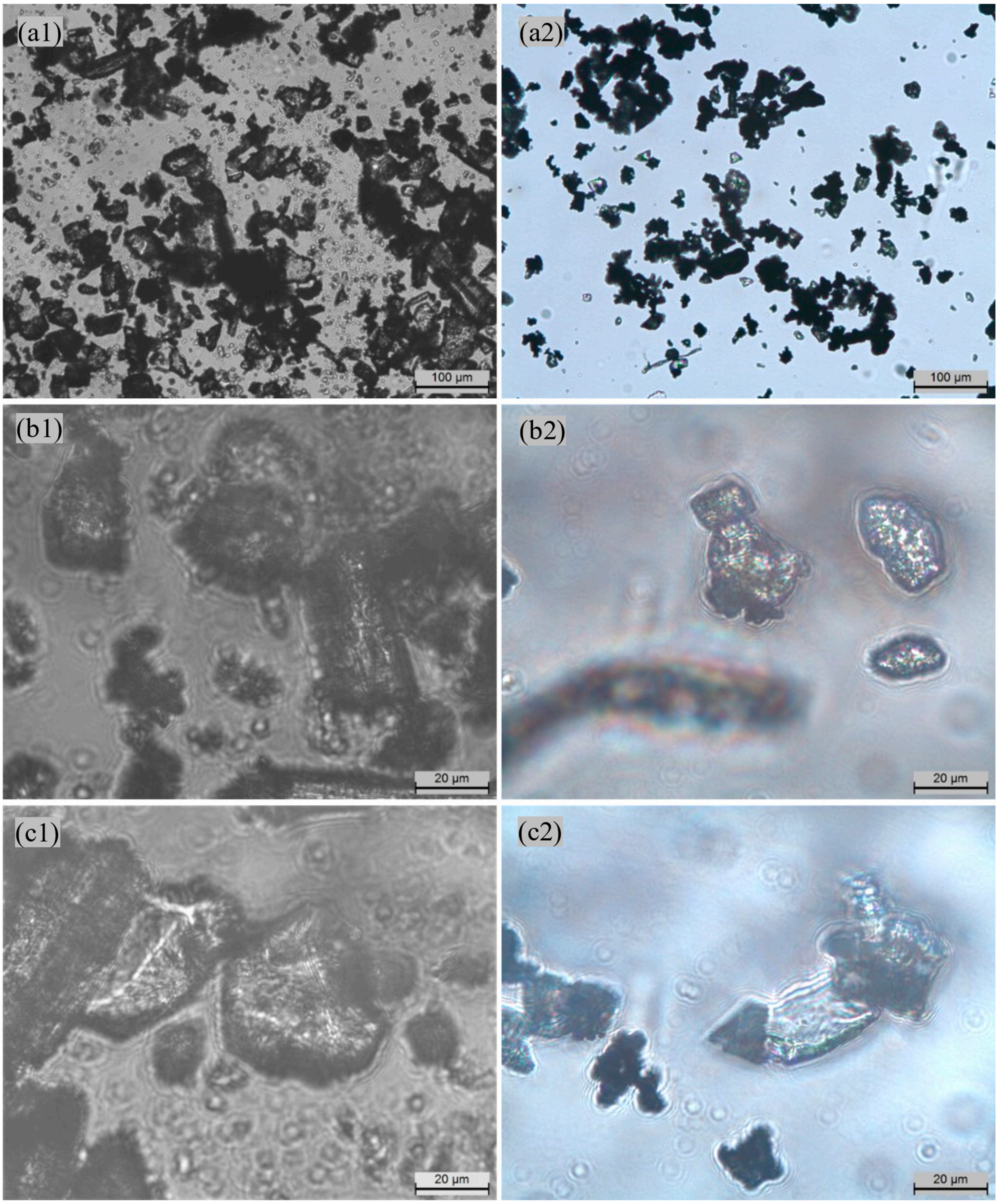

Figure S2. Microscopic images taken for particle size determination of Porcelite (**a1**), (**b1**) and (**c1**), and Ferrolite (**a2**), (**b2**) and (**c2**).

Scanning electron microscopy of printed samples

The layer-by-layer structure of the printed samples using G-Strong, Porcelite and Ferrolite resins was seen in SEM images. For G-Strong and Porcelite, the layer thickness, z_{print}, was set as 50 µm. For Ferrolite, the layer thickness was set as 40 µm. For this thickness, the corresponding values exposure time was set as 1.9, 3.6 s and 3.8 s for G-Strong (**Error! Reference source not found.**a), Porcelite (**Error! Reference source not found.**b) and Ferrolite (**Error! Reference source not found.**c), respectively. The analysis is performed on a Vega TS5136 XM Tescan microscope (Tescan Brno s.r.o., Kohoutovice, Czech Republic). Prior to SEM analysis, samples were gold-sputtered.

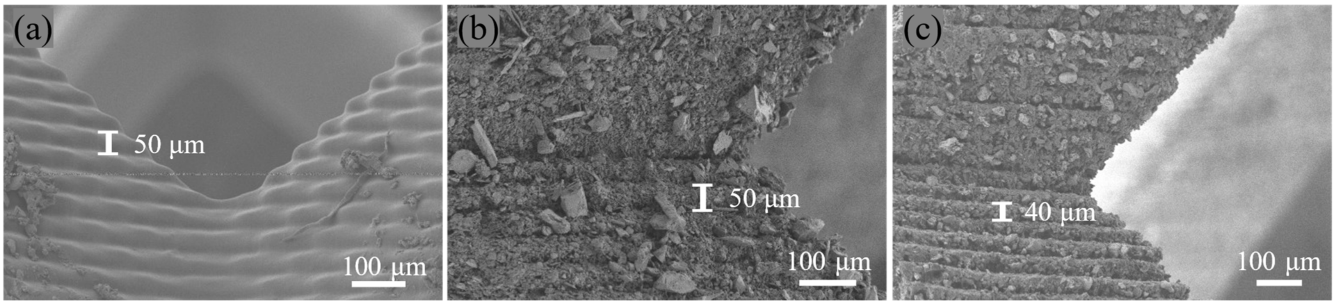

Figure S3. SEM images showing the layer-by-layer structure of G-Strong (**a**), Porcelite (**b**) and Ferrolite (**c**).

Printing of the samples outside the printing window

For further validation of the printing space, samples were printed at the lower limit of the printing space, t_{min}: 0.8 s for G-Strong, 0.9 s for Porcelite and 0.3 for Ferrolite. However, the base layer was exposed at the optimized time of printing, which was used to print samples (as demonstrated in Figure 7) to ensure the adhesion to the print head, for respective resins: 1.9 s for G-Strong, 3.6 s for Porcelite and 3.8 s for Ferrolite. The upper limit, t_{max}, was not considered for the printing to avoid over-polymerization of the printing layers that could result in damaging the vat base after few detachments. **Error! Reference source not found.**a shows the partial detachment of the printing sample base using G-Strong. However, complete printing failure was observed for Porcelite (**Error! Reference source not found.**b) and Ferrolite (**Error! Reference source not found.**c) as the exposure time was not sufficient to ensure good adhesion of the base layer to the print head, which was however optimum for adhesion between the layers.

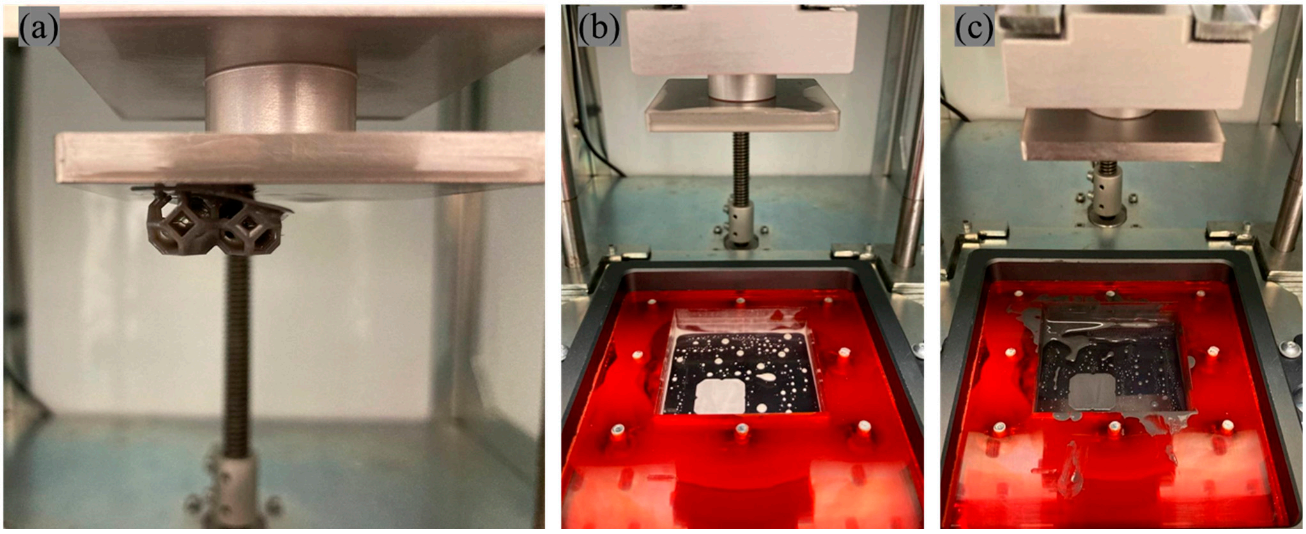

Figure S4. Printing of the samples outside the printing space. The sample printed with G-Strong showed detachment from the print head during printing (**a**). Also, a reduction of exposure time for Porcelite (**b**) and Ferrolite (**c**) did not allow the base layers to adhere to the print base.
